# Explanatory models of post-traumatic stress disorder (PTSD) and depression among Afghan refugees in Norway

**DOI:** 10.1186/s40359-021-00709-0

**Published:** 2022-01-04

**Authors:** Dixie Brea Larios, Gro Mjeldheim Sandal, Eugene Guribye, Valeria Markova, David Lackland Sam

**Affiliations:** 1 Department of Psychosocial Science, University of Bergen, Christies Gate 12, Postboks 7807, 5015 Bergen, Norway; 2 NORCE Research, Kristiansand, Norway; 3 Department of Pulmonology, Haukeland University Hospital, Bergen, Norway

**Keywords:** Refugees, Explanatory models, PTSD and depression, Afghan refugees

## Abstract

**Background:**

The current situation in Afghanistan makes it likely that we are facing a new wave of Afghan refugees, warranting more knowledge about how to deal with mental health problems among them. This study aims to gain more knowledge on Explanatory Models (EM) of depression and post-traumatic stress disorders (PTSD) among Afghan refugees resettled in Norway.

**Methods:**

We conducted six gender-separated, semi-structured focusgroup interviews based on vignettes with Afghan refugees (total N = 27). The vignettes described a fictional character with symptoms of either depression or PTSD symptoms in line with DSM-5 and ICD-10 criteria.

**Results:**

The findings showed that EM varied with gender, age, generation, and migration stories. Participants suggested different potential causes, risk factors, and ways of managing symptoms of depression and PTSD depending on the context (e.g., in Norway vs. Afghanistan). In describing the causes of the depression/PTSD in the vignettes, females tended to emphasize domestic problems and gender issues while males focused more on acculturation challenges. The younger males discussed mostly traumatic experiences before and during flight as possible causes.

**Conclusion:**

The practice of condensing a single set of EMs within a group may not only be analytically challenging in a time-pressed clinical setting but also misleading. Rather, we advocate asking empathic questions and roughly mapping individual refugee patients’ perceptions on causes and treatment as a better starting point for building trusting relationships and inviting patients to share and put into practice their expertise about their own lives.

**Supplementary Information:**

The online version contains supplementary material available at 10.1186/s40359-021-00709-0.

## Background

While many groups of refugees have consistently shown high prevalence of mental health problems [1–4], underutilization of mental health services, alternative help-seeking preferences, and different illness explanatory models have consistently posed barriers for effective treatment [5–7]. Earlier research has documented variations in the way refugee groups explain and view mental health problems [8, 9]. Discrepancies in understanding conceptions of mental health may hamper the recognition of mental health problems in patients from other societies, with the risk of misdiagnosis or treatment failure.

The current situation in Afghanistan in the fall of 2021 makes it likely that we are facing a new wave of Afghan refugees, warranting more knowledge about how to deal with mental health problems among them. In this article, we investigate explanatory models (EMs) of posttraumatic stress disorders (PTSD) and depression among Afghan refugees settled in Norway. We argue for a need to venture beyond notions of static cultural models, since culture is often characterized by intra-cultural variations (differences within a cultural group related to e.g., geography, education, gender and occupation); a dynamic interplay between individual agency and social processes; and renegotiations of cultural understandings and interpretations with others [10, 11]. Thus, we propose a need to improve our understanding and identify how refugee groups perceive and prefer to cope with mental health problems in dynamic, fluid, and multiple ways.

### Explanatory models

The concept of illness explanatory models (EMs) has been employed to ensure culturally sensitive care, enhance therapeutic alliances between professionals and patients, and improve our understanding of help-seeking paths, treatment compliance, and receptivity to health promotion messages [12–14]. A string of seminal works in medical anthropology throughout the 1970’s (e.g., [15–17]) led to the recognition that patient-doctor interactions are transactions between EMs that may often diverge from each other in terms of explanations, expectations and goals [18]. Kleinman [19] defined EMs as notions about an illness episode and the treatment employed by those involved in a clinical process, including patients, their families, and their doctors. With the aid of a series of qualitative questions, Kleinman attempted to bring to light variations in beliefs about causes for a patient’s illness and ideas about treatment and outcomes, as a clinical tool.

Finding that differences in perceptions about symptoms and treatment may cause patients to underutilize public health services or drop out of treatment [5, 20, 21], a great deal of research has focused on mapping explanatory models among various ethnic groups, refugees, and migrants [8, 22–24]. Many studies in this field have found that mental health problems such as depression and PTSD are often perceived as resulting from psychosocial causes rather than physical or biological [24], and that participants in these studies often prefer to cope with their problems by relying on e.g., family, social networks, traditional healers, and religious practices, rather than professional treatment inside public health services.

The concern that a lack of understanding of perceptions of mental health problems in low-income and war-torn countries may represent a barrier towards effective treatment for refugees, has led to a revision of the *Diagnostic and Statistical Manual of Mental Disorders* (DSM-5). Consequently, in DSM-5 *cultural concept of distress* is used to distinguish cultural traits of mental health experiences including cultural explanations or perceived causes, as well as cultural idioms of distress and cultural syndromes [25]. Nevertheless, being able to map and identify clear-cut models of people’s explanations of illnesses and preferred ways of treating them, is still problematic. Kleinman [19] maintained that EMs among patients, their families, and doctors may each be multiple. Subsequent research found that EMs may consist of a variety of often contradictory explanations that may be held at the same time, rather than a coherent set of beliefs [11, 26, 27]. Kleinman [28] argued against the formalism, specificity, and boldness of explanatory models. The idea that people are equipped with static illness-related templates has been challenged by studies that have found considerable intra-cultural differences, for instance, differences related to geography, education, gender, and occupation [29–32].

Furthermore, perceptions of illness and treatment preferences have been found to be transient and inconsistent among patients across time [33], and may vary according to social context, i.e., person presenting different EMs at home, at work, and in the doctor’s office [11]. Studies have also found that professional biomedical EMs may influence popular ideas about illness in several ways, making unclear distinctions between professional and lay person’s EMs [34]. Moreover, positive experiences from encounters with public mental health services, or beliefs and practices in the country of settlement, may improve the use of public mental health services [7, 35]. Consequently, there is a need for a better understanding of how these kinds of dynamic processes may also influence EMs among refugees.

### Explanatory models among Afghan refugees

In the current study, we explore explanatory models of PTSD and depression among Afghan refugees resettled in Norway. We investigate variations in EMs related to gender and age and pose the question of how processes of migration and acculturation may influence the Afghan EMs of depression and PTSD. Research has consistently shown high prevalence of, and comorbidity between PTSD and depression in refugee populations, including Afghans [2, 4, 36–41]. A survey conducted in Afghanistan suggested that one out of two Afghans (50%) is suffering from psychological distress and one out of five (20%) is impaired in his or her role because of mental health problems [42]. The report concluded that Afghan people are very much exposed to trauma and PTSD is frequent in contrast to other disorders such as clinically significant major depression disorders or generalized anxiety [42].

Studies among Afghan refugees resettled in various parts of the world report a variety of ways to cope with mental health challenges, including confronting stressors, helping others, social support, focus on the future, religion, exercise, avoidance, positive thinking, and professional help, among others, although informal help-seeking seems to be preferred [5, 43–46]. The same variation applies to attributed causes of stress. For instance, a study among Afghan refugees in New Zealand found that sources of stress included factors such as cultural clashes, resettlement issues, economic concerns, lack of trust, separation from family, homesickness, lack of work, and worry about family in war zones [45].

However, although populations of Afghan refugees have steadily increased in Western countries in the recent decades, there is a paucity of studies on EMs among Afghans related to specific diagnoses such as PTSD and depression. A study among Afghan refugees in California found that respondents perceived that depression, often expressed as *afsurdagi* (Dari for grief, low mood, and sadness), was caused by a variety of factors including traumatic experiences, cultural adjustment challenges and conflicts, interpersonal challenges, uncertainty about the future, loss of identity and having chronic diseases [29]. The study also found gender divergences, in which women tended to associate depression with more somatic items [29]. Furthermore, a study in Australia suggested poor understanding of mental health symptoms, differences in health care information and treatment practices between Afghans and the mainstream population [47]. Another study in Australia on causes of, and risk factors for PTSD showed that experiencing traumatic events, coming from a war-torn country, as well as family problems were cited as causes among Afghan refugees [48]. In summary, these studies suggest that EMs among Afghan refugees are multiple, divergent, and influenced by traumatic experiences, migration, and acculturation factors, rather than being a static, coherent set of cultural beliefs.

The number of Afghan refugees in Norway has tripled since 2006 [49]. Recent research shows that 20.4% of Afghan refugees in the country reported mental health problems in the form of depression and anxiety [39]. Moreover, Afghan refugees are among the minority groups reporting most problems related to solitude in Norway [50]. Although mental health services have improved in this millennium, they are still sparse in Afghanistan [51–53]. However, many of Afghan refugees may have had previous experiences with mental health services in for example refugee camps, influencing their EMs about PTSD and depression. Moreover, all newly arrived refugees in Norway also enroll in an obligatory full-time introductory program for up to 2 years, which often includes a focus on health and mental health, as well as public health services in the country.

To this background, the current study aimed to investigate EMs of depression and PTSD among Afghan refugees in Norway. Considering previous research, which suggest that EMs are fluid, multiple, and subject to change according to social context, we aimed to gain more knowledge about potential intra-cultural variations in the EMs (specifically related to gender and age), and to invite our participants to reflect on the extent to which their EMs may have changed because of the migration to another country.

## Methods and materials

### Participants

The study included six gender-separated focus group interviews (FGIs) with two to six participants and one individual interview (since the remaining participants did not show up for that FGI). Participants were recruited from the 2-year compulsory introductory program for refugees in three municipalities in Norway and adult educational programs. The inclusion criteria were that they were refugees from Afghanistan, above the age of 18, and had lived more than 6 months in Norway. A minimum of 6 months residence time in Norway was chosen as a cut-off because it takes up to 6 months for newly arrived refugees to formalize their settlement status and get full access to Norwegian education and health services. Staff in the programs facilitated the recruitment by identifying and inviting potential participants, providing them with information about the study. As part of the process, suitability for participating in group interviews with a focus on mental health was considered, based on the possible risks that discussions could bring about significant discomfort and sensitive topic. There was no mental health assessment of participants prior to participation in the FGIs.

A total of 27 Afghan refugees between the ages of 18 and 47 (15 females and 12 males) resettled in Norway participated in this study. Table 1 presents information about the groups. Most of the participants had a spouse from Afghanistan. Except for two participants who had Pashto as their main language, the native language of participants was Dari. The duration of stay in Norway ranged from 1 to 14 years. Table 2 shows the demographic characteristics of the participants.

**Table 1:** Focus group participants (N = 27)

Focus group	Age range	Gender	Group size	Vignette character
1	19–42	Female	4	PTSD
2	18–20	Male	5	PTSD
3	35–37	Male	4	Depression
4	25–30	Female	5	Depression
5	31–47	Male	2	Depression
6	29–37	Female	6	Depression
Individual interview	34	Male	1	PTSD

**Table 2:** Socio-demographic characteristics for focus group participants (N = 27). Missing data are due to no response from the participants on some of the characteristics. Two female participants were both employed and currently studying in Norway

Socio-demographic characteristics	Males	Females	Total
	N = 12	N = 15	N = 27
*Age*			
18–25 years	5	4	9
26–30 years		4	4
31–35 years	3	2	5
36–40 years	3	4	7
41–45 years		1	1
46–50 years	1		1
*Current situation*			
Employed	-	2	2
Unemployed	-	13	13
Student (attending Norwegian language course)	7	2	9
*Family status*			
Married	6	13	19
Single	5	–	5
Other (separated, divorced, widowed)	1	-	1
*Educational level*			
Less than high school	1	4	5
High school diploma	5	2	7
College degree or higher	2	-	2
*Language*			
Dari	11	14	25
Pashto	1	1	2

### Procedure

The focus group interviews took place during February, March, and August of 2019. The FGIs were conducted using a vignette displaying a fictional person suffering from symptoms of either PTSD or depression in line with ICD-10 (*Classification of Mental and Behavioral Disorders*) and DSM-5 (see Additional file 1: Appendix) [25, 54]. The gender of the vignette character was matched to the participants (males or females) to facilitate identification. The vignettes were adapted from previous research on explanatory models among refugees [24, 47, 55, 56]. The vignettes were translated from English to Dari and Pashto by a professional translation service and back-translated to the Norwegian by interpreters participating in the focus group interviews. The interviews took place within the facilities belonging to the municipality (i.e., library, classroom, and facilities of the introductory program). Licensed interpreters of the same gender as the participants attended the interviews. Questions were asked in Norwegian and translated to Dari or Pashto by the interpreters. Most participants had basic Norwegian language skills and could choose to answer in Norwegian or in their native language. The interpreters translated the answers given in Dari or Pashto into Norwegian and helped clarify potential misunderstandings.

Before the interviews, the interpreters explained their role and assured confidentiality. The interpreters read aloud the consent agreement in the native language of the participants, making sure that everyone understood the content. Next, the interpreters read the vignettes out loud in the native language of the participants (Dari or Pashto). After eliciting the groups’ initial responses and thoughts about the situation of the vignette character, the major follow-up questions were: What, if anything, do you think is wrong with Mossa/Zarina? What could be the reason Mossa/Zarina is feeling the way he/she does? If you were his/her friend, what would you recommend him/her to do? And why? Does he/she have a disease? Do you think Mossa/Zarina can get help from the public health sector? If yes, how? If not, why not? Do you think there are differences in how people in Afghanistan and Norway think about this situation?

Closed questions were followed up with more open questions. The interviewers urged free discussion, still making sure that all groups covered the main topics, that all members were to some degree active, and that the discussion was centered. The participants were never encouraged to reveal personal information but were asked to imagine that they were friends of the vignette character. The interview guide was based on previous research [19, 57], and further developed in cooperation with an Afghan research associate.

Three members of the research team (authors one, two, and four) were present during the interviews. Authors two and four, both clinical psychologists, conducted the interviews. The interviews lasted 1.5–2 h and were video- and audio-recorded. No compensation was given to the participants. Coffee, tea, and snacks were served during the interviews.

### Ethics

The data collection was approved by the Norwegian Regional Ethical Committee (Reference number: 2018/1794 and 2018/2115), and the Norwegian Center for Research Data (Reference number: 602214). All methods used were performed in accordance with the relevant guidelines and regulations. Before the interviews started, the participants were orally informed about the purpose of the study, that participation was voluntary, how data would be handled in all phases of data collection and publication, and that confidentiality would be protected. They were also informed that they could leave the interview at any time without explaining the reason. All participants signed a consent agreement in Dari or Pashto before the interviews started and were encouraged not to reveal information that had been shared with other participants during the interviews to other people. One participant was excluded from the interview due to being underage. One FGI became an individual interview as the other invited participants did not show up.

### Data analysis

The interviews were transcribed verbatim in Norwegian, masking the identity of the participants, and reviewed by the first author and a research assistant. All transcriptions from the focus group interviews were coded and analyzed following the principles of thematic analysis [58]. All authors coded data separately to improve truthfulness, searched for and identified themes marking up suitable categories both manually and with the software program NVivo [59]. The analysis focused on causal explanations, ideas about coping, and treatment options associated with PTSD and depression. Codes were identified, developed into broader categories, and thematically clustered. Quotes were selected to describe the themes and each theme was explored and organized by gender. The quotes selected had been translated from Dari and Pashto to the Norwegian language during the interviews and transcription. The quotes presented in this article were translated into the English language by the research team. Several themes emerged from each of the main categories, sometimes unique for either the PTSD or depression interviews.

## Results

We have organized the findings of explanatory models of PTSD and depression among Afghan refugees in Norway into two sections, separated by gender: (1) Themes related to causes and risk factors; and (2) Themes related to managing symptoms. Where relevant, we will discuss differences related to vignette type (PTSD or depression).

### Causes and risk factors

#### Females

Female participants used the terms *depression* (for both the depression and PTSD vignettes), *stress* (depression vignette), and *trauma* (PTSD vignette) in both Norwegian and English languages to describe the condition of the vignette character. Several of them furthermore expressed that they recognized the situation of the vignette characters from their personal experience: “I have the same problem as Zarina, I carry the same story. I experienced the same.”

The females emphasized that the described symptoms may derive from several causes depending on the person or social context. For instance, one of them said:

It varies from person to person. Some have problems. For instance, they have problems with their families, with their children, they are sick themselves. When children are sick, they are concerned all the time about the one who is sick. When you are concerned all the time, you naturally start to forget yourself. You think it is a difficult everyday life. Perhaps they […] experienced war, it varies from person to person. And perhaps some might have problems with their husband, their home in their family.

The generally unstable and insecure situation in Afghanistan was also considered a cause of distress after resettling in Norway: “It says here [referring to the vignette], that she has family from Afghanistan. Maybe she lost somebody in her family in Afghanistan. It is not safe there. There has been a war there, and it is terrible there. Maybe she thinks about it.”

Despite this emphasis on multiple possible causes depending on the context, a repeated theme was how the vignette character’s symptoms could relate to gender issues. Several plausible causes discussed, included forced marriages, often at a young age (e.g*. *“She must have been forced to marry this man”); gender roles (e.g. “They are not allowed to go out without family or relatives”); domestic violence (e.g. “Most of them in Afghanistan have experienced a situation in their lives …domestic violence… if they have faced that in their home country, obviously, they’re going to feel like Zarina”)*;* harassment and violence against women (e.g. “If she gets pregnant out of wedlock, she will be rejected by her family, and she will be killed. She will be seated in the middle of a room in front of men, and they will throw stones at her”); and social control and generational conflicts (e.g., “All the time the parents must decide”)*.* Participants also emphasized that these types of causal factors could depend on whether the woman came from urban or rural areas in Afghanistan*.*

The practice of social control as a source of distress for women could also continue after resettling in Norway: “Maybe mom and dad say that she must wear hijab, or she disagrees with her parents. That she had to wear a hijab, or she had a boyfriend and wanted to marry a man she wanted.” One woman commented that a likely reason for the depression of the vignette character was that her parents had not given her sufficient insight into what is culturally appropriate among Afghans. The participants noted that the vignette character with depression lived in a different city from her parents and that her distress could be related to guilt (e.g., “She has a guilty conscience. She can't help her parents”) and feeling disconnected (e.g., “I think she has lost contact with her parents”). Work overload could exacerbate feelings of social isolation. One commented: “She’s worked for 5 years and now she can’t take more work. She has lost sleep as well. She has no time for friends or her sister.”

#### Males

Similar to the female participants, males associated the vignette character’s problems with their own and stressed that the problems may have several causes. However, different themes emerged compared with the women, and there were also differences between the depression and PTSD vignettes. While the females emphasized gender-related issues regarding both vignettes, the males in the depression FGIs emphasized acculturation challenges, while the younger men in the PTSD FGI, emphasized experiences before, and during flight, and the concerns associated with being an asylum-seeker.

Some of the participants in the depression groups identified the vignette character's condition as *depression.* However, they had several notions of *depression.* Participants noted that mental health problems differ from somatic diseases (e.g., “There is when you are ill physically, and then there are other ways, when you are depressed, you are mentally ill”), and that conditions of depression may differ in terms of severity: (e.g., “I thought he [Mossa, the vignette character], was a little depressed. Not so deep, in the first stage. In that condition, we can quickly help these people. If we don’t help them quickly it can get worse.” Importantly, several participants also commented that depression is a condition not observed in Afghanistan, since people there often have what they considered more serious mental health problems due to trauma and the hardships of living:

In Afghanistan, I would say we don’t have this issue. If somebody has depression, we do not have it in Afghanistan. I am not very conservative or cautious, but why don’t we have it? Because many are in shock. Many cannot carry on. We have many psychological problems, but they are not because of depression.

The groups recognized that depression is reflected in cognitive patterns of self-blaming and faultily attributing negative events to personal inadequacies. For instance, one commented: “If I have a friend, and then today he is not friends with me, maybe it is because I was a bad person before [referring to the way of thinking of depressed people]. I believe that it is not true because all people have problems, but some can distance themselves.” One group emphasized that a traditional view among Afghans is that problems or poor health are somehow deserved. One said: “We think if you are a bad person then everything follows. If you have an allergy, that is because you're a bad person, for example. It is the culture that we have. The old, especially older people think a lot like that.” Religious beliefs could also impose feelings of guilt contributing to depression according to some participants, but none of the participants endorsed spiritual explanations to mental health problems [e.g., being a bad Muslim].

Consistent with the view that depression mostly emerges after resettling, many of the possible causes discussed were challenges associated with acculturation. One participant mentioned: “It is not just him that is under pressure. It is all refugees who are in this country. They are under pressure because of the language, lack of work, communication problems, and so on.” Financial problems in exile were also highlighted as contributing to mental health problems. Loneliness and feelings of being disconnected were mentioned by several of the men recognizing that the vignette character was unmarried and lived alone, e.g. “Most Afghans who have problems here, it is because they live alone” and “they cannot explain the problems they have. They just have it inside them and that makes them depressed.”

It was emphasized that cultural confusion could amplify mental health problems. One mentioned that young men could have problems related to how they approach Norwegian women because they carry with them gender norms from the Afghan culture:

Because of low education or not having an education in the home country, they have not read about this country or laws. So, for example, if he sees a girl without a scarf in his home country, he thinks she is a whore or something. And then he thinks that those who are here are exposed in the same way. And when afterwards he sees that no, it is not like that here, and he will be depressed.

Intergenerational and family conflicts due to acculturation challenges were also mentioned, e.g., “Because children who are born in this country, they want the same culture as their [Norwegian] friends. And there may be a little crash and constantly there may be arguments with the parents.” Another cause associated with the adaptation to a new country included the harsh climate in Norway: “He should go on a journey because in the Norwegian climate you can quickly become sad.”

The younger participants in the PTSD group identified the vignette as related to trauma (“It is mental problems, trauma”). The discussion centered on experiences before, and during the flight as causal factors; and stressors after resettlement as exacerbating or maintaining PTSD symptoms. Comments about the vignette character often intermeshed with their personal accounts. Memories about violence, war and fear could continue to haunt the vignette character after resettling in Norway. One commented:

I don’t like to watch social media. Many things happen in our home country that we can watch immediately on TV or social media. Bombing and people dying happen almost every day. It is painful to see and listen to. Maybe it is my family who is dying. He [the vignette character] has experienced it. Something which has impacted him.

One of the participants, referring to his own experience said: “People are afraid that when you’re asleep, people will come and kill you.” The group discussed how encounters during flight could cause these kinds of symptoms from the vignette character. One commented that the vignette character’s symptoms may have been caused by “What he has experienced there [on the way from Afghanistan to Norway]. This may continue in his thoughts and lead to the problems that he has in Norway.” Several mentioned feeling vulnerable and unprotected during flight, e.g., that the symptoms may have been exacerbated by “injections and some drugs he may have been given […] perhaps somebody wanted him to develop mental problems.” After arriving in Norway, the possibility of having asylum applications rejected and being returned to Afghanistan was also mentioned as a stressor: “Lately there have been many rejections. He might be forced to return. Many Afghans are returned. So, it could be because of many rejections, and [what is written] in the media about it.” Mental health problems were also considered to be amplified by rumination related to the atrocities and difficult living conditions faced by people in Afghanistan: “He can for instance think about poor people, think about people there and that there has been a war for so long.”

### Managing symptoms of depression and PTSD

#### Females

The females’ discussions about managing symptoms of PTSD and depression were relatively similar. As with causes and risk factors, it was emphasized that ways of managing symptoms depended on the context, with a major distinction between the situations of women in Norway and Afghanistan. A repeated theme was that women in Afghanistan have no other choice than persisting living under very difficult conditions, such as domestic violence. One woman said: “She will just tolerate the burden by herself.” Help-seeking sources outside the family were of little use:

I heard there are organizations, but they are not working. They agree when [family members from both] families are there, but when the wife is back to her husband's house, she is facing the same problem. There is no way. So, she will be facing it until she is dead.

Also, according to the participants, admitting to emotional problems in Afghanistan would run a risk of being branded “*crazy*,” an attitude that would hold them back from seeking help.

Talking to a trusted person emerged as the primary advice they would give to the vignette character independent of whether she had symptoms of depression or PTSD and whether she is in Norway or Afghanistan. Clearing her mind of negative thoughts and feelings was an essential part (e.g., “It often helps to tell others what someone has in their heart… it helps a lot, and one could feel relieved”). Talking to one’s husband, a family member, or friend was the first choice (e.g., “If she has a family, a sister or a brother, or the oldest daughter, she could talk to close relatives”)*.*

However, situations were discussed in which the woman could not talk to other Afghans due to taboos about mental health problems, shame, or cultural practices, e.g., an unmarried woman having slept with a man. In those instances, turning to a doctor, psychologist, or other health professionals were seen as particularly helpful, since they have a duty of confidentiality and are not permitted to disclose information. Also, otherwise, most of the women thought that a psychologist could help Zarina, e.g. “I think she needs to talk to a psychologist. Empty her heart. Everything she has inside must come out.” However, some women objected and commented that psychologists often only offer medication in form of treatment. One of the females had personal experience from seeing a psychologist in Norway after losing her parents and concluded that “Psychologists do not help. Only family and friends.”

Furthermore, emphasizing the role of family for positive mental health, getting married and starting a family was seen as a good way for the unmarried vignette character to manage symptoms of depression (e.g., “She needs to find a good man"; “She needs to find a boyfriend so there is a change in her life”). Several commented that becoming pregnant and having another child (for married women) could help to regain hope for the future and keeping the mind busy and away from difficult thoughts and emotions. Other ways of managing the symptoms were also considered as potentially useful depending on the assumed cause of the condition of the vignette character. When being over-worked was suggested as a possible explanation, finding time for herself (e.g., “She needs to think about herself.”)*,* going on holidays, enjoying more time and relaxation with friends, and keeping herself busy were important measures.

#### Males

Similar to the female participants, the males presented with the depression vignette, highlighted that managing the symptoms depended on the context. When discussing the possibility that the causes could be linked with isolation and loneliness, measures such as reconnecting with family and socializing with friends were highlighted as essential. One male mentioned how visiting the home country had led to a profound transformation in a friend suffering from depression. Also, as in the female FGIs, males discussed getting married to overcome the situation (e.g., “If he is in need to get married, he must get married or find someone in his life. Most Afghans have problems here because they live alone”).

Regardless of what caused the symptoms, talking to family or a friend they trust, were seen as  the first step towards treatment and recovery. One of the most important forms of support provided by a friend could be to guide the person to seek help from a GP, who could help him recognize he has a problem. One of the males talked about having been prescribed medication for a similar condition to the vignette character. Nonetheless, barriers for contacting public health care system were also discussed, including that a 15-min appointment would be too short for the GP to understand the problem; lack of trust (e.g., that childcare services would take away their children); and that most psychologists lack an adequate understanding of the background of Afghan refugees.

Some also discussed other ways of managing the symptoms of depression, including religion (e.g., “Maybe religion would have helped him”), setting goals (e.g., “He should have a goal in life or find one”), and having a job, like the vignette character. Thus, the participants in the depression FGIs did not prescribe a single set of responses and help-seeking options but suggested multiple ways that the vignette character could deal with his problems.

The young men in the PTSD group emphasized that engaging in distracting activities such as sports, hiking with friends, or having a job could alleviate feelings of distress. Avoiding revoking memories or being exposed to upsetting information by e.g., limiting use of social media was seen as essential measures. One mentioned how it could be helpful to release difficult emotions; “He can go to another place where he can get that aggression out… Scream or something. Get everything out. It helps to relax.”

While participants recognized that not everyone wants to talk about their problems, sharing thoughts and feelings about past experiences was seen as vital to overcome symptoms of PTSD. One participant commented:

Talking to others and not being alone, perhaps not being too much on social media, seeing a psychologist helps when you need it. We think that's also very important. And not carrying too much inside and using the opportunities to talk to someone and how you feel - and if you go to think about things for yourself, then you get a little bit, you don't feel better right? You just feel worse. So, I think it is very important to share what you must carry. And don't hide it inside. Too often it doesn't go away.

The young men in the PTSD FGI had arrived in Norway as unaccompanied minors who did not have family in the country. Some of them described difficulties in developing friendships with Norwegian youth at their age: “*When I went to junior high school, I was very happy to make Norwegian friends, but …. they did not talk much to me…, so eventually I lost interest.”* The gap in experience was also seen as a barrier in talking to Norwegians: “*It is difficult for Norwegians to understand such problems. Because they do not have them. And we Afghans understand*.” Thus, turning to friends, family, or other people from Afghanistan was considered most helpful as they could most easily identify with and understand the situation. However, they also reflected on the fact that sometimes this help was not available to them, and that some of them had sought and received valuable support from adult professionals such as social workers, teachers, or psychologists who had become part of their lives in Norway in one way or another.

## Discussion

Our study is the first to examine explanatory models of depression and PTSD among Afghan refugees in Norway, building on previous studies, which suggest that EMs are fluid, multiple and subject to change according to social context [19, 29, 60]. The findings supported our expectations that EMs are characterized by intra-cultural variations based on factors such as gender and age. All participants stressed that there might be many potential causes, risk factors, and ways of managing symptoms of depression and PTSD depending on the context. To the extent that we found recurring themes, they varied among distinct categories of participants, e.g., with females emphasizing domestic problems and gender issues as possible causes, in contrast with males who tended to emphasize acculturation challenges and loneliness. The perceived association between depression and resettlement was salient in that several males claimed that depression hardly exists in the Afghan context. The younger males focused most on traumas experienced before, and during flight in their causal interpretation.

Furthermore, participants maintained that there are different options for managing symptoms in Afghanistan and Norway, e.g., with females discussing how the situation of the vignette character would have to be endured in Afghanistan, while in Norway, there were other ways of managing the symptoms. Nonetheless, although women recognized that they have more freedom of choice in the Norwegian context, gender-based social control persisted even after resettlement in Norway that contributed to symptoms of depression.

The findings of our study share both similarities and differences with previous studies among Afghan refugees across the world (see [5, 43–46]. An inclination towards a preference for informal help sources seems to be a shared finding across studies, also those involving other minority groups [55, 61]. Likewise, the emphasis on the value of family as an institution converges with findings from other studies [22, 29]. For example, participants noted that moving away from parents and remaining umarried at the age of 27 (referring to the vignette character) could amplify mental health problems due to guilt and feeling disconnected. Findings such as poor understanding of mental health symptoms among Afghan refugees in Australia [47], or the tendency among Afghan refugee women in California to associate depression with more somatic items [29], could not be replicated in our study. Rather, most interview groups quickly identified the condition of the vignette character as depression, and it was commented that depressed individuals typically view undesirable occurrences in life as having internal, stable causes. This is in line with contemporary research on cognitive patterns associated with vulnerability to depression [62]. Thus, overall, our study supports the notion that it is misleading to think about EMs as a single set of coherent cultural explanations (that may be applicable for refugee groups from the same nation across countries of settlement) [19].

One of the inherent problems with EMs as a concept, is that there is a risk for a too strong focus on (often exotic) cultural practices at the expense of understanding the influence of social context [14]. The perception that illness needs to be understood within the perspective of the forms of treatment available within a cultural system initially attracted empirical studies in cultural contexts where the interplay between shamans, oracles, orthodox medicine, divinations, bonesetters, herbalists, practitioners of western medicine and others came into focus [19]. However, more recent studies have found that mental health disorders are more often attributed to social factors such as material living conditions and relationships than to spiritual or supernatural factors [63, 64]. Similarly, our study suggests that the social context, i.e., separation from family, domestic relationships, war, flight, and resettlement challenges, seems to have contributed strongly to shape the EMs of the Afghan refugees in Norway. For instance, in line with previous studies among unaccompanied minors in Norway [65], the focus group consisting of young men arriving in Norway as unaccompanied minors stressed that lack of Afghan friends and family, and difficulties in building friendships with Norwegian youths creates a situation in which talking to professional adults (e.g., social workers, psychologists and medical doctors) is the best alternative. Likewise, for the females, turning to health care professionals was seen as particularly useful when experiencing problems that could be difficult to discuss with family and social network due to taboos or cultural norms. The implication is that the EMs of these refugees seems to reflect a realistic assessment of treatment options available to them in the current social setting in exile, more than a cognitive model of cultural perceptions brought with them from the country of origin [55]. It should be recognized that some of the coping patterns described by the young men in the PTSD focus group are general characteristic for people exposed to trauma, including avoidance of emotional experiences and situations that bring about reminders of trauma. Thus, withdrawing from social media as described by these young men should be seen as a normal response to highly distressing life experiences.

Overall, how the refugees interpret and prefer to manage mental health problems are influenced by their encounters with Western mental health systems and services throughout the migration experience. Several of them used the term depression to describe the condition of the vignette character although a simple translation of this word is not available in Dari or Pashto (the native languages of the participants). Despite the dearth of mental health services in Afghanistan [53], many of the refugees had previous experiences with NGOs offering mental health services during, and after flight. They had learned about the Norwegian health system during the compulsory introduction program for newly arrived refugees and they had experiences from personal consultations with the Norwegian health system, including psychologists and GPs. Clearly, these experiences, along with acculturation challenges and other migration-related experiences had contributed to shaping their EMs. Again, this observation supports the need for more focus on the relationship between social processes and cultural practices and perceptions [9, 14].

However, an assessment of the validity of our findings is warranted. Although participants, to a varying degree, were conversant in Norwegian, much of the discussions between them were in their native language (mostly Dari). In terms of cultural validation, the Afghan interpreters and a research assistant from Afghanistan gave cultural insight during the process including commenting on the relevance and validity of the vignettes to Afghan refugees settled in Norway. On the other hand, possible bias associated with the use of interpreters needs recognition. The quality of the translations was vital for the obtained data, and any inaccuracies or misapprehensions may possibly have resulted in errors in our interpretations [66, 67]. We acknowledge that the interactions between the Afghan refugees and professional psychologists moderating the FGIs may have involved transactions between EMs in its’ own right, in that the participants deliberately downplayed more “exotic” cultural perceptions in favor of a more “western” explanatory framework. This kind of power issue comes into play in research interview settings in general [68]. Our participants based on our perception, hardly discussed any rich, “exotic” cultural palette of causes and treatment options. They also seemed reluctant to discuss the role of religion in much depth. With the current anti-Islamic rhetoric in the public discourse, this is understandable. However, participating in a focus group interview about their views and experiences contributed to create a setting that invited reflections and reassessments of what causes mental health problems and how to deal with them. Expressing that they identified with the vignette character, further supports the trustworthiness of the findings from the study.

This is further strengthened by the fact that the participants also discussed EMs in rural Afghanistan, contrasting them with their own perceptions of living in exile in Norway. This kind of process is also consistent with contemporary understandings that culture involves a dynamic interplay between individual agency and social processes, renegotiations of cultural understandings, and interpretations of cultural meanings with others [10]. Studies have found that with acculturation, the EMs of ethnic groups evolve and move towards the majority culture [69], which corroborates with the present study. However, more research is warranted on the influence of migration experiences and social context on refugee EMs.

One possible bias of this study is that we did not have a balanced sample of participants in terms of age, education, and family situation in groups discussing PTSD and depression vignettes. The participants in the male focus group discussing the PTSD vignette were younger (between the ages of 18–21) high school students, and had arrived in Norway as unaccompanied minors. Also, they were single, while members of the other groups were married, except for one male. Furthermore, the limited size of the sample warrants follow-up quantitative studies to identify more categorical patterns. Future research may consider eliminating some of the two vignette characters' background differences that could potentially influence interpretations beyond the mental health symptoms presented. Specifically, the PTSD vignette character had a flight background, was married, and had children, while the depression vignette character was single, and no information about immigrant background was given (although the character had a common Afghan name). Notably, the present study did not include a clinical sample based on diagnosed psychopathology. The high incidence of depression and PTSD among refugees suggests that a large proportion will either experience these conditions themselves or within their family. Past research [56] indicates that in communal societies, family members are influential for health services utilization and treatment preference. Thus, we argue that the views of lay people might be informative for how the Afghan refugees deal with mental health problems.

This study has important clinical implications. With the inclusion of cultural concepts of distress in DSM-5, the importance of dealing with potential divergences in the EMs of patients and professionals is recognized, and several instruments for mapping EMs have been developed across the years (for an overview, see [70]). However, in clinical practice these methods are seldom applied due to time constraints and unfamiliarity with social science methods among many professionals such as psychiatrists [70, 71]. We advocate that the idea of systematically developing an explanatory *model* in the sense of condensing a single set of causal explanations and treatment preferences may not only be analytically challenging in a time-pressed clinical setting but also misleading [27]. The empirical diversity evident in our study and others may make it more appropriate to talk about *maps* rather than *models* [19].

The concept of models may be too static to convey the fluid status of perceptions among patients. There are risks that tentative and uncertain perceptions explored by individuals may become reified and made definitive in the shape of a coherent explanatory model [27]. Our study demonstrates that there is not a single accurate and generalized explanatory model representing Afghan perceptions on causes and treatment, and that individual perceptions are likely to continue to change across time. A map in the shape of a snapshot in time of various avenues of thought and less fixed possibilities may provide a more relevant framework for exploring options with the patient.

Kleinman [28] maintained that he aimed to establish a method to promote clinical self-reflexivity and improve clinical communication using open-ended questions and negotiations Our participants’ perceptions about depression and PTSD as related to trauma and challenging life circumstances did not deviate from how natives or health professionals could think about such diseases. They did not indicate that these mental health diseases were associated with stigma. Yet, the participants had reservations about the ability of professionals to understand the situations of Afghan refugees. By asking empathic questions and roughly mapping the patients’ perceptions, professionals may have a better starting point for building trusting relationships with refugee patients and invite them to share and put into practice their expertise in their own lives.

## Supplementary Information

**Additional file 1**. PTSD and depression vignettes. The FGIs used a vignette displaying a fictional person suffering from symptoms of either PTSD or depression in line with ICD-10 and DSM-5.

## Data Availability

The data are available from the corresponding author on reasonable request.

## Abbreviations

FGI: Focus group interview
EM: Explanatory models
DSM-5: Diagnostic and statistical manual of mental disorders
ICD-10: International classification of diseases and related health problems
