# Supplementary material for: Explanatory models of post-traumatic stress disorder (PTSD) and depression among Afghan refugees in Norway
Source: BMC Psychol. 2022 Jan 4;10:5. doi: 10.1186/s40359-021-00709-0 (PMC8728976; doi:10.1186/s40359-021-00709-0)
Supplement: Supplementary file 1 — Additional file 1. PTSD and depression vignettes. The FGIs used a vignette displaying a fictional person suffering from symptoms of either PTSD or depression in line with ICD-10 and DSM-5. [file 40359_2021_709_MOESM1_ESM.docx]

# **Appendix**

The PTSD-vignette read as follows:

*Mossa/Zarina is a 27-year-old waiter in a restaurant in Bergen. He/she was born in Afghanistan but had to flee his/her home country. Mossa/Zarina lives together with his wife/her husband and children. When he/she first came to Norway, he/she felt relieved and thought about the future. He/she made plans for himself and his family. However, soon he/she is troubled by frequent nightmares about experiences that he had before and during the flight, he/she has problems falling asleep, and he/she wakes up many times every night. He/she often experiences flashbacks of frightening memories, and avoids conversations, situations, for example TV-programs, that can remind him/her of difficult experiences. He/she avoids places that awaken memories from the past that he/she tries to forget. He/she often feels tense and has pain in his body without any reason. He/she is easily startled when hearing loud sounds such as a car backfiring or fireworks. Lately both his wife/her husband and colleagues in the restaurant have commented that he/she seems restless and irritable. He/she feels numb and indifferent to people around him/her, and even has difficulties with having positive emotions when he is together with his children. Mossa/Zarina has tried to become more engaged in his/her family and work, but he/she finds it very hard. This makes him/her feel guilty and worthless. It is important for him/her to take care of his/her children. When asked about how he/she feels about the future, Mossa/Zarina replies that he/she feels hopeless and does not believe he/she will live a long life.*

The depression-vignette reads as follows:

*Mossa/Zarina is a 27-year-old waiter in a restaurant in Bergen. He/she was born in Oslo to parents who were restaurant owners but has made Bergen his/her home for 5 years. In the last few weeks, he/she has been experiencing feelings of sadness every day. Mossa/Zarina's sadness has been continuous, and he/she cannot attribute it to any specific event or to the season. It is hard for him to go to work every day; he/she used to enjoy the company of his/her co-workers and working at the restaurant, but now he/she cannot find any pleasure in this. In fact, Mossa/Zarina has little interest in most activities that he/she once enjoyed. He/she is not married and lives alone, near his/her brother/sister. Usually, they enjoy going out together and with friends. But now he/she does not enjoy this anymore. Mossa/Zarina feels very guilty about feeling so sad and feels that he/she has let down his/her brother/sister and friends. He has tried to change his/her work habits and start new hobbies to become motivated again, but he/she cannot concentrate on these tasks. Even his/her brother/sister has now commented that Mossa/Zarina gets distracted too easily and cannot make decisions. Since these problems began, Mossa/Zarina has been poorly sleeping every night; he/she has trouble falling asleep and often wakes up during the night. A few nights ago, as he/she lay awake, trying to fall asleep, Mossa/Zarina began to cry because he/she felt so helpless.*
